# Turning as a single and dual task—associations with Alzheimer’s and vascular pathologies in cognitively healthy older people

**DOI:** 10.1093/gerona/glag114

**Published:** 2026-05-07

**Authors:** Maria H Nilsson, Magnus Lindh-Rengifo, Lynn Rochester, Danielle Van Westen, Ruben Smith, Sebastian Palmqvist, Erik Stomrud, Niklas Mattsson-Carlgren, Oskar Hansson

**Affiliations:** Department of Health Sciences, Faculty of Medicine, Lund University, Lund, Sweden; Clinical Memory Research Unit, Department of Clinical Sciences Malmö, Faculty of Medicine, Lund University, Lund, Sweden; Memory Clinic, Skåne University Hospital, Malmö, Sweden; Department of Health Sciences, Faculty of Medicine, Lund University, Lund, Sweden; Memory Clinic, Skåne University Hospital, Malmö, Sweden; Translational and Clinical Research Institute, Faculty of Medical Sciences, Newcastle University, Newcastle upon Tyne, United Kingdom; Diagnostic Radiology, Clinical Sciences Lund, Lund University, Lund, Sweden; Image and Function, Skåne University Hospital, Lund, Sweden; Clinical Memory Research Unit, Department of Clinical Sciences Malmö, Faculty of Medicine, Lund University, Lund, Sweden; Memory Clinic, Skåne University Hospital, Malmö, Sweden; Clinical Memory Research Unit, Department of Clinical Sciences Malmö, Faculty of Medicine, Lund University, Lund, Sweden; Memory Clinic, Skåne University Hospital, Malmö, Sweden; Clinical Memory Research Unit, Department of Clinical Sciences Malmö, Faculty of Medicine, Lund University, Lund, Sweden; Memory Clinic, Skåne University Hospital, Malmö, Sweden; Clinical Memory Research Unit, Department of Clinical Sciences Malmö, Faculty of Medicine, Lund University, Lund, Sweden; Memory Clinic, Skåne University Hospital, Malmö, Sweden; Clinical Memory Research Unit, Department of Clinical Sciences Malmö, Faculty of Medicine, Lund University, Lund, Sweden; (Medical Sciences Section)

**Keywords:** Postural balance, Wearable sensor, Amyloid, tau, White matter hyperintensities

## Abstract

**Background:**

Turning performance is an important and challenging motor activity, with potential relevance in several brain diseases. This study aimed to determine how Alzheimer’s and vascular pathologies are associated with turning (360° while standing and 180° while walking), both as a single task and during dual-tasking in cognitively healthy older people.

**Methods:**

Cognitively healthy older people (*n* = 297, mean age 77.7 years, 61.6% women) were included. A 180° turn was assessed using a wearable sensor; a 360° turn was assessed both clinically and using a sensor. Dual-tasking included a simultaneous subtraction task. Alzheimer’s pathologies (Aβ and tau) were assessed using positron emission tomography (PET). Vascular pathology (white matter hyperintensities (WMH)) was assessed using magnetic resonance imaging (MRI). Logistic and linear regression analyses were used.

**Results:**

Aβ and tau pathologies were significantly associated with being unstable while dual-tasking and turning 360°. When including both Aβ and tau in the model, only tau remained significantly associated with being unstable (OR 22.42, 95% CI, 1.43, 349.88, *p* = .027). WMH were associated with impaired turning as a single task, that is, an increased turn duration (360°) and a decreased peak angular velocity (180°: B −1.27, 95% CI, −2.33, −0.21, *p* = .018).

**Conclusions:**

This study suggests that early Alzheimer’s pathologies are associated with an instability while turning 360° and dual-tasking, whereas WMH seem to be associated with impaired turning as a single task in cognitively healthy older people. Turning assessments seem promising for investigating motor aspects in very early stages of Alzheimer’s disease and vascular brain disease.

## Introduction

The development of brain pathologies precedes cognitive symptoms and a diagnosis of mild cognitive impairments (MCI) or a major neurocognitive disorder (ie, “dementia”). Alzheimer’s and vascular pathologies are the main causes of progressive cognitive impairment in older people. These pathologies are associated with gait impairments in older people without dementia.^1^^,^^2^ Moreover, decreased gait speed precedes cognitive decline,^3^^,^^4^ and those with decreased motor function seem to have a faster cognitive decline.^5^ It is therefore of interest to gain an increased understanding of early motor signs in relation to neurocognitive disorders, including their associations with early brain pathologies. If interested in the early stages, it is valuable to address cognitively healthy older people, that is, as brain pathologies precede cognitive impairments. As turning is a more challenging motor task than walking straight ahead,^6–8^ it may be more sensitive to early stages of brain pathologies than walking. Despite this, there is a lack of studies that assess how turning performance relates to Alzheimer’s and vascular pathologies.

Turning is both a complex motor task and a frequent activity in everyday life,^9^^,^^10^ and turning performance declines with increasing age.^8^^,^^11^^,^^12^ Among older people without dementia, turning has been associated with cognitive functioning, eg, executive functioning,^13^ processing speed,^7^^,^^14^ memory,^9^^,^^14^ and visual-spatial abilities.^7^^,^^9^ The difficulty of turning seems to increase with a greater turning angle.^8^^,^^15^ A motor task becomes more difficult when adding a cognitive task, that is, “dual-tasking.” In relation to gait and mobility, a mental tracking task (eg, subtraction) appears to be the most sensitive cognitive task for detecting MCI.^16^ Future dual-task research has been recommended to explicitly focus on turning as the motor task.^17^

Alzheimer’s disease is the most common cause of dementia, and it is characterized by amyloid-β (Aβ) and tau pathology. Aβ pathology is commonly measured by using the cerebrospinal fluid (CSF) Aβ42/40 ratio or by using positron emission tomography (PET).^18^ These Aβ-biomarkers show high agreement.^19^^,^^20^ Tau pathology can be measured using CSF phosphorylated tau (p-tau) or tau-PET.^21^ Cerebral small vessel disease (especially white matter hyperintensities, WMH) is instead a manifestation of vascular pathology, which is common in older people and possible to detect and quantify with magnetic resonance imaging (MRI).^22^

Increased levels of WMH have been associated with decreased gait speed and falls.^1^^,^^23^ Moreover, low motor functioning has been associated with a larger WMH volume and a smaller total brain volume.^5^ Amyloid pathology is common in hip fracture patients^24^ and associated with falls in preclinical Alzheimer’s disease.^25^ Among cognitively unimpaired old people, Aβ has also been associated with impaired gait and balance.^26–29^ Although several studies have addressed Alzheimer’s pathology in relation to mobility^27^^,^^30–34^ (ie, Timed Up & Go [TUG], which includes turning), there is limited knowledge of how Alzheimer’s pathologies relate to the specific task of turning, that is, in standing or while walking.

Among cognitively healthy older people, we anticipated that early Alzheimer’s pathologies would primarily be associated with 360° turns while dual-tasking, that is, as this is considered more complex than turning as a single task or than making a smaller turn.

This study aimed to determine how Alzheimer’s and vascular pathologies are associated with turning (360° while standing and 180° while walking) both as a single task and during dual- tasking in cognitively healthy older people.

## Method

### Participants

Participants in this cross-sectional study were part of the BioFINDER-2 study’s (www.biofinder.se, NCT03174938) cohort of cognitively healthy older people (*n* = 300).^35^ This cohort also conducts detailed motor assessments, which are part of the sub-study Motor-ACT. However, one participant was missed, rendering a sample size of 299.

Older community-dwelling people were recruited by using random sampling from two prior population-based community cohort studies, see Supplemental material (Methods).

The BioFINDER-2 study had several inclusion and exclusion criteria for this cohort. Inclusion criteria were as follows: age 66-100 years, not fulfilling the criteria for MCI or dementia according to the DSM-5,^36^ a Mini-Mental State Examination (MMSE)^37^ score of 26-30 points at the screening visit, absence of cognitive symptoms (as assessed by a physician), and an interpreter was not required for the participant to understand the study information and perform cognitive testing. The physician’s assessment of cognitive function was based on cognitive testing, medical history, and data based on the following instruments: the Brief Anosognosia Scale (BAS), the Cognitive Function Instrument (CFI), and the Cognitive Impairment Questionnaire (CIMP-QUEST). The participants were also assessed with more detailed and thorough cognitive assessments, see Supplemental material (Methods).

Exclusion criteria were as follows: significant unstable systemic illness or organ failure (eg, terminal cancer) that makes it difficult to participate in the study; current alcohol/substance misuse, refusing lumbar puncture, MRI or PET; and significant neurological (eg, Parkinson’s disease) or psychiatric illness.

The current study had an additional exclusion criterion, that is, missing PET data (tau or Aβ), *n* = 2.

Ethical approval was obtained by the Regional Ethical Review Board in Lund (2016-1053) and the Swedish Ethical Review Authority (2019-02681). All participants provided written informed consent before the start of the study.

### General procedure of data collection

The data collection began on January 1, 2018, but wearable sensors were not implemented until October 2019. Consequently, fewer participants had sensor data than clinical turning data. Alzheimer’s pathologies (Aβ and tau) were assessed using PET. Vascular pathology (WMH) was assessed using MRI. Supplemental material (Methods) clarifies the time between assessments.

### Turning assessments

Turning assessments were performed by specifically trained physical therapists at the Memory Clinic, Skane University Hospital. The physical therapist provided oral instructions and demonstrated each test.

All participants performed the turning assessments in the same order: a 180*°* turn while walking was followed by turning 360*°* in standing. The turns were initially assessed as single tasks, and thereafter, while dual-tasking (ie, by using a subtraction task). No instruction was given on whether the participant should prioritize the motor and/or the cognitive task. Assessments were initiated by the command “start now.”

Regarding the cognitive subtraction task (ie, as a single task in sitting and while dual tasking), the following was registered: the total number of subtractions, correct subtractions, and the time. See supplementary material for details (Methods, and STable 1).

#### Turning 180° while walking

Turning 180° was assessed during the TUG test, which includes several tasks: to rise from a chair (45-46 cm in height and with armrests), walk 3 m, turn 180°, walk back and turn around to sit down again. A small cone (5.5 cm high, with a base diameter of 19 cm) visualized where the participant had walked 3 meters. In this study, we only report the first turn in the TUG. Using mobility devices was allowed, but physical support by a person was not allowed.

TUG was conducted in two test situations: A) comfortable pace, and B) dual-tasking, that is, comfortable pace while serially subtracting 3 (start at 100) by counting out loud.

Between the two test situations, participants conducted the cognitive task, that is, serial subtractions by 3 (start at 30) for 10 seconds in a seated position. While dual-tasking, the subtraction task was initiated after the commando (ie, in sitting) and continued throughout TUG.

The total time of TUG (s) was registered by using a digital stopwatch, see Table 1. Turning 180° while walking was assessed by using a wearable sensor (turn peak velocity and turn duration), see further below.

**Table 1:** Characteristics of cognitively healthy participants, descriptive values, and missing data, *N* = 297.

	Mean (*SD*) unless otherwise stated	Missing, *n*
**Age (years)**	77.7 (5.3)	–
**Sex (women), *n* (%)**	183 (61.6)	–
**BMI (kg/m^2^)**	26.6 (3.9)	–
**Education (years)**	12.2 (3.7)	–
**History of falls- past year (yes), *n* (%)**	74 (24.9)	–
**Fear of falling (yes), *n* (%)**	84 (28.7)	4
**Global cognitive functioning (MMSE)**	28.7 (1.2)	–
**Cognitive functioning (composite score, mPACC5), median (q1-q3)**	−0.23 (−0.71, 0.22)	1
**A history of stroke/TIA (yes), *n* (%)**	21 (7.1)	–
**Cardiac history (yes), *n* (%)**	24 (8.1)	–
**Comfortable gait speed (m/s)**	1.2 (0.18)	3
**Mobility (Timed up & go—single task/dual task, s)**	10.3 (2.1)/13.7 (4.3)	−/2^a^
**Amyloid-β (PET, SUVR)**	0.53 (0.13)	–
**Tau (PET, SUVR, Braak stage I-IV)**	1.19 (0.12)	–
**White matter hyperintensities (MRI and mL)**	4.52 (5.52)	4
**Intracranial volume (mL)**	1502.1 (156.5)	2^b^
**Clinical assessments of turning 360° in standing (both directions)**	Single task/dual, *n* = 297	
Unstable (= scored 0-3), *n* (%)	114 (38.8)/137 (48.9)	3^c^/17^d^
Timed, both turns, stopwatch (s)	6.7 (1.5)/9.8 (3.4)	3^c^/17^d^
**Sensor data of turning 360° in standing (both directions)**	Single task/dual, *n* = 180	
Peak angular velocity (degrees/s)	207.9 (44.5)/167.2 (41.4)	2^e^/11^f^
Turn duration, both turns (s)	6.5 (1.3)/9.2 (2.4)	2^e^/11^f^
**Sensor data of turning 180° while walking**	Single task/dual, *n* = 180	
Peak angular velocity (degrees/s)	141.3 (31.7)/120.0 (29.2)	−/2^a^
Turn duration (s)	3.0 (0.5)/3.3 (0.6)	−/2^a^

The use of sensors was implemented later. Reported percentages are valid percentages. Abbreviations: BMI, body mass index; MMSE, Mini-Mental State Examination (scoring range: 0-30, higher = better); MRI, magnetic resonance imaging; mPACC5, modified preclinical Alzheimer’s cognitive composite score; TIA, transient ischemic attack; PET, positron emission tomography; SUVR, standardized uptake value ratio.

a Two did not manage the test while dual-tasking.

b These two also had missing data on white matter hyperintensities.

c Three had missing data: 2 needed a mobility device, 1 did not manage the test.

d Two needed a mobility device, 14 did not manage the test, and 1 was missing.

e One needed a mobility device, 1 did not manage the test.

f One needed a mobility device, 7 did not manage the test, and 3 had missing data.

#### Turning 360° in standing (both directions)

The participant was standing and instructed to turn around 360°, stop and then turn around 360° in the other direction. No instructions were given regarding whether to start turning to the left or right. Using mobility devices was not allowed, which also applied to physical support by a person.

The test was conducted in two situations: A) turning 360° (both directions) as a single task, and B) turning 360° (both directions) while dual-tasking, that is, the participant was also doing serial subtractions by counting out loud (−3, start at 99).

One trial was conducted for test A and B, respectively. If the participant did not manage the test, a second trial was conducted, including an oral prompt at the end of the first turn: “Stop and turn around in the other direction.” There were no instructions regarding which pace to use.

For each of the two test situations, the total time (seconds, 2 decimals) for turning 360*°* in both directions was recorded by using a digital stopwatch. The performance was also scored and assessed by using a wearable sensor (ie, turn duration and turn peak velocity, see below). Balance performance was scored from 0-4: 0 = loses balance so that the assessor must physically intervene; 1 = takes more than 1 compensatory step; 2 = takes 1 compensatory step; 3 = takes no compensatory steps, but has an increased postural sway, or 4 = good balance with no compensatory steps or increased postural sway. If hesitant between two different scoring options, the assessor recorded the worst one. Those who scored from 0 to 3 were classified as unstable (coded 1), and those scoring 4 were classified as stable (coded 0).

### Wearable sensors

The APDM Mobility Lab System (Opal^®^ V2 Solutions, APDM Wearable Technologies [Portland, Oregon, USA], a Clario Company) was used, which includes six wearable and wireless sensors. Each sensor includes an accelerometer (3 axes), gyroscope (3 axes), and magnetometer (3 axes). The sample rate was 128 Hz. The sensors were placed according to the APDM Mobility Lab User Guide. By using data from the lumbar sensor (posterior placed at the L5 level), turning parameters (180° and 360° turns, ie, as single tasks and while dual-tasking) were extracted with the APDM Mobility Lab software. The following parameters were extracted: highest registered turn peak velocity (degrees/s) and turn duration (s). For the 360° turn, we used the total turn duration of turns 1 and 2, which required creating a new variable.

Further clarification: all registered turns were extracted. If fewer than two turns had been recorded when collecting sensor data, these data were not included. If more than two turns had been registered, raw sensor data were inspected along with video recordings of the test for each participant. The data were recalculated to capture the 1st and 2nd turns of each test scenario. This was performed in both single and dual task scenarios and applied to both the TUG test and the 360° turn test.

### PET and MRI

Tau and Aβ pathology were assessed using PET.^38^ Tau PET imaging ([^18^F]RO-948) was performed 70-90 minutes after injection of ∼370 MBq of the radiotracer. Standardized uptake value ratio (SUVR) was calculated, using the inferior cerebellar cortex as a reference region. A volume-weighted composite temporal meta-ROI (ie, entorhinal cortex, inferior and middle temporal cortices, fusiform gyrus, parahippocampal cortex, and amygdala) corresponding to Braak Stages I-IV^39^ was created using FreeSurfer software (v.6.0).

Amyloid-β PET imaging ([^18^F]flutemetamol) was performed 90-110 minutes post-injection of ∼185 MBq of the radiotracer. A composite neocortical meta-ROI for Aβ aggregates was created (FreeSurfer v.6.0), including prefrontal, lateral temporal, parietal, anterior cingulate, and posterior cingulate/precuneus ROIs, using the pons as reference region.

SUVR measures were used as continuous measures in the analyses.

MRI scans were performed using a Siemens 3T MAGNETOM Prisma scanner (Siemens Healthineers, Erlangen, Germany). By using an open-source toolbox (ie, lesion segmentation tool) implemented in SPM8, a total white matter lesion volume (labelled WMH, mL) was generated.^40^

### Covariates

The selection of adjusting factors was based on prior studies, which showed an association with both brain pathologies and motor aspects/or dual-tasking, for example, education.^41^

Adjusting factors included age (years), biological sex (women = coded 1), a history of stroke/TIA (yes = coded as 1), and education (years).

### Additional variables

Additional descriptive data included the following: body mass index (BMI, kg/m^2^), MMSE, and mobility (TUG in comfortable pace, sec.), see Table 1.

Variables that reflected frailty were also included: cognitive functioning, cardiac history (including both ischemic heart disease and congestive heart failure, yes = coded as 1), a history of falls (past year, yes = coded as 1), fear of falling (yes = coded as 1), and comfortable gait speed. See Table 1 for descriptive information. In relation to cognitive functioning, a composite z-score was used, that is, a modified Preclinical Alzheimer’s Cognitive Composite 5, mPACC5:^42^ ([the Alzheimer's Disease Assessment Scale, ADAS, delayed recall x 2] + animal fluency + MMSE + Symbol Digit Modalities Test [SDMT]) / 5. All cognitive test scores were initially transformed into z-scores, that is, before the mPACC5 calculation. Comfortable gait speed (single task) was assessed by performing at least six trials (continuous walking) on an electronic walkway (GAITRite® platinum, CIR Systems Inc., Franklin, New Jersey, USA).

### Analyses

Descriptive data are presented as mean (*SD*) or frequency (percentage).

In regression analyses, four turning parameters constituted the dependent variables. Logistic regression analyses (Odds ratios [OR], 95% CI, *p*-values) were used for the dichotomous dependent variable that related to instability (unstable: coded 1) while turning 360° in standing. Linear regression analyses were used for the remaining three turning parameters, that is, turn duration (timed and sensor) and peak angular velocity (sensor). Unstandardized regression coefficients (B) are presented with 95% CI, including *p*-values.

Independent variables (WMH, tau, and Aβ) were initially tested for multicollinearity (no correlation surpassed a threshold of ±0.7).

Regression analyses were done in 4 steps. Steps 1-3 included all dependent variables, where each model only included one brain pathology (Aβ, tau, or WMH).

*Step 1)* A series of univariable (ie, unadjusted) regression models.

*Step 2)* Adjusted for age and sex. WMH were also controlled for intracranial volume (ICV), which applies to all the following steps that include WMH. Steps 1 and 2 are presented in STables 2-5.

*Step 3)* Adjusted for age, sex, a history of stroke/TIA, and education, see Tables 2 and 3.

**Table 2:** Multivariable regression analyses (linear or logistic, each pathology was adjusted for age, sex, stroke/TIA, and education) with different turning 360° parameters as dependent variables in cognitively healthy older people.

	Turning 360° in standing- single task			
	Timed task, stopwatch (s) *n* = 294	Unstable (coded 1) *n* = 294	Peak angular velocity, sensor (degrees/s) *n* = 178	Turn duration, sensor (s) *n* = 178
	B (95 % CI) *p*-value	OR (95 % CI) *p*-value	B (95 % CI) *p*-value	B (95 % CI) *p*-value
Aβ PET, SUVR	−0.15 (−1.42, 1.12) *p* = .815	2.84 (0.44, 18.09) *p* = .269	10.92 (−35.80, 57.63) *p* = .645	−0.19 (−1.49, 1.12) *p* = .778
Tau PET, SUVR	1.15 (−0.22, 2.51) *p* = .098	4.01 (0.48, 33.03) *p* = .197	−1.70 (−70.24, 66.83) *p* = .961	−0.21 (−2.12, 1.71) *p* = .833
WMH, mL	0.04 (0.01, 0.08) ***p* = .018**	1.01 (0.96, 1.06) *p* = .650	−1.33 (−2.84, 0.18) *p* = .084	0.05 (0.01, 0.10) ***p* = .016**

	Turning 360° in standing− dual tasking			
	Timed task, stopwatch (s) *n* = 280	Unstable (coded 1) *n* = 280	Peak angular velocity, sensor (degrees/s) *n* = 169	Turn duration, sensor (s) *n* = 169
Aβ PET, SUVR	2.16 (−0.86, 5.17) *p* = .160	10.08 (1.30, 77.70) ***p* = .027**	−5.19 (−50.83, 40.45) *p* = .823	−1.36 (−1.22, 3.94) *p* = .297
Tau PET, SUVR	1.80 (−1.40, 5.00) *p* = .268	37.24 (2.64, 523.79) ***p* = .007**	−2.04 (−68.69, 64.62) *p* = .952	1.40 (−2.37, 5.17) *p* = .462
WMH, mL	0.04 (−0.04, 0.12) *p* = .297	1.05 (0.99, 1.11) *p* = .073	−0.91 (−2.33, 0.51) *p* = .205	0.07 (−0.02, 0.15) *p* = .130

Models including WMH were also adjusted for intracranial volume. In models that included WMH, 3 and 4 had missing data. Abbreviations: Aβ, amyloid-β; B, unstandardized regression coefficient; CI, confidence interval; OR, odds ratio; PET, positron emission tomography; SUVR, standardized uptake value ratio; TIA, transient ischemic attack; WMH, white matter hyperintensities (assessed by using magnetic resonance imaging).

Aβ PET SUVR was according to a neocortical meta region of interest (ROI): prefrontal, lateral temporal, parietal, and anterior cingulate and posterior cingulate/precuneus, using pons as the reference region. A composite temporal meta-ROI was used for tau pathology (ie, entorhinal cortex, inferior and middle temporal cortices, fusiform gyrus, parahippocampal cortex, and amygdala), using the inferior cerebellar cortex as reference region. The stability variable (original scoring 0-4, higher = better) was dichotomized as follows: unstable (scores 0-3, coded as 1) and good balance/stable (score 4, coded as 0). Statistically significant values are bolded.

**Table 3:** Multivariable linear regression analyses (each pathology was adjusted for age, sex, stroke/TIA, and education) with different turning 180° parameters as dependent variables in cognitively healthy older people.

	Turning 180° while walking (sensor)			
	Single task	Dual tasking	Single task	Dual tasking
	Peak angular velocity (degrees/s) *n* = 180	Peak angular velocity (degrees/s) *n* = 178	Turn duration(s) *n* = 180	Turn duration(s) *n* = 178
	B (95 % CI) *p*-value	B (95 % CI) *p*-value	B (95 % CI) *p*-value	B (95 % CI) *p*-value
Aβ PET, SUVR	2.77 (−30.37, 35.90) *p* = .869	−8.12 (−39.20, 22.96) *p* = .607	−0.04 (−0.62, 0.54) *p* = .896	0.18 (−0.47, 0.83) *p* = .589
Tau PET, SUVR	19.93 (−28.40, 68.25) *p* = .417	−8.31 (−53.75, 37.12) *p* = .718	−0.01 (−0.86, 0.84) *p* = .984	−0.03 (−0.92, 0.98) *p* = .954
WMH, mL	−1.27 (−2.33, −0,21) ***p* = .018**	−0.78 (−1.77, 0.21) *p* = .121	0.02 (−0.01, 0.04) *p* = .068	0.00 (−0.02, 0.03) *p* = .687

Models including WMH were also adjusted for intracranial volume. In models that included WMH, 3 had missing data. Abbreviations: Aβ, amyloid-β; B, unstandardized regression coefficient; CI, confidence interval; PET, positron emission tomography; SUVR, standardized uptake value ratio; TIA, transient ischemic attack; WMH, white matter hyperintensities (assessed by using magnetic resonance imaging).

Aβ PET SUVR was according to a neocortical meta region of interest (ROI): prefrontal, lateral temporal, parietal, anterior cingulate and posterior cingulate/precuneus, using pons as the reference region. A composite temporal meta-ROI was used for tau pathology (ie, entorhinal cortex, inferior and middle temporal cortices, fusiform gyrus, parahippocampal cortex, and amygdala), using the inferior cerebellar cortex as reference region. Statistically significant values are bolded.

Based on the findings of step 3, the subsequent step focused on dependent variables that showed a statistically significant association with more than one brain pathology.

*Step 4)* The brain pathologies were simultaneously entered with adjusting factors: age, sex, stroke/TIA, and education, see Table 4. This was followed by sensitivity analyses, see Table 4 including its footnotes, and Tables (S6A-C).

**Table 4:** Multivariable logistic regression analysis with instability during 360° turning and dual-tasking as the dependent variable in cognitively healthy older adults. Each model included both Aβ and tau, and all models were adjusted for age, sex, stroke/TIA, and education.

	Turning 360° in standing- being unstable while dual tasking (coded 1)		
	Original model	**Sensitivity analysis (excl. 2 outliers tau-PET)** ^a^	**Sensitivity analysis (excl. 2 outliers tau-PET + adjusting for cognition)** ^b^ ^,^ ^c^
	*n* = 280	*n* = 278	*n* = 278
	OR (95 % CI) *p*-value	OR (95 % CI) *p*-value	OR (95 % CI) *p*-value
Aβ PET, SUVR	4.62 (0.53, 39.73) *p* = .164	4.62 (0.53, 39.57) *p* = .163	4.88 (0.55, 42.91) *p* = .153
Tau PET, SUVR	22.42 (1.43, 349.88) ***p* = .027**	19.61 (1.10, 348.94) ***p* = .043**	20.20 (1.12, 362.61) ***p* = .041**

Abbreviations: Aβ, amyloid-β; CI, confidence interval; OR, odds ratio; PET, positron emission tomography; SUVR, standardized uptake value ratio; TIA, transient ischemic attack.

The dependent variable (original scoring 0-4, higher = better) was dichotomized as follows: unstable (scores 0-3, coded as 1) and good balance/stable (score 4, coded as 0).

Aβ PET SUVR was according to a neocortical meta region of interest (ROI): prefrontal, lateral temporal, parietal, anterior cingulate and posterior cingulate/precuneus, using pons as the reference region. A composite temporal meta-ROI was used for tau pathology (ie, entorhinal cortex, inferior and middle temporal cortices, fusiform gyrus, parahippocampal cortex, and amygdala), using the inferior cerebellar cortex as reference region.

Cognition: a modified preclinical Alzheimer’s cognitive composite (mPACC5) was calculated as follows: ([the Alzheimer's Disease Assessment Scale, ADAS, delayed recall x 2] + animal fluency + MMSE + Symbol Digit Modalities Test [SDMT]) / 5. Statistically significant values are bolded.

a If also adjusting for cardiac history, the results were as follows. Aβ PET 4.44 (0.51, 38.14), *p* = .174, Tau PET: 20.11 (1.12, 359.17), *p* = .041, *n* = 278.

b If adjusting for comfortable gait speed instead of cognition, the results were as follows. Aβ PET: 4.49 (0.52, 38.82), *p* = .172.

Tau PET: 22.43 (1.20, 418.85), *p* = .037, *n* = 275. Tau remained significant if also adjusting for cardiac history: 22.63 (1.21, 422.85), *p* = .037.

c If adjusting for a history of falls instead of cognition, the results were as follows. Tau PET: 19.39 (1.09, 344.63), *p* = .043, *n* = 278. Tau remained significant if also adjusting for cardiac history: 19.85 (1.11, 354.09), *p* = .042.

Sensitivity analyses adjusted for variables that can reflect frailty, which has been associated with both decreased motor functioning and Alzheimer’s pathology.^43^^,^^44^ We initially adjusted for cognition (mPACC5), but we also considered cardiac history, a history of falls, and comfortable gait speed. An additional sensitivity analysis included only those without fear of falling and a history of falls.

For multivariable linear regression analyses, residuals were assessed visually for normality, linearity, and homoscedasticity. In relation to multivariable logistic regression analyses, the Hosmer and Lemeshow test was used for model validation; the model fits the data if the *p*-value exceeds .05.

Statistical analyses were performed using the software IBM SPSS Statistics, version 29 (IBM Corp., Armonk, NY, USA).

## Results

The final sample included 297 cognitively healthy older persons (mean age 77.7 years, *SD* 5.3; 61.6% women; 21 had a history of stroke/TIA; mean years of education 12.2, *SD* 3.7). Descriptive data and missing data are presented in Table 1. Sensors were implemented later, and 180 out of 297 had sensor data.

Regarding the 360° turn, two participants did not complete the test due to being dependent on using a mobility device. As a single task, one participant (1/295, 0.34%) did not manage the test. Moreover, 18/294 (6.1%) required a second trial to manage the test. While dual-tasking, one participant had missing data, and 14/294 (4.8%) did not manage the test. Out of the 280 remaining participants, 87 (31%) required a second trial to manage the test.

While dual-tasking, a higher proportion was unstable: 38.8% (single task) versus 48.9% (dual-tasking), see Table 1. Moreover, both the motor and cognitive tasks worsened, see STable 1.

Regarding the 180° turn, all participants managed the test as a single task, whereas two did not manage it while dual-tasking.

All unadjusted analyses are presented in the supplemental material, see STables 2 and 4.

### Turning 360° in standing as a single task: associations with brain pathologies

Four dependent variables were used in the regression analyses that targeted turning 360° (ie, as a single task and while dual-tasking): being unstable while turning (clinically assessed), turn duration (stopwatch), turn duration and peak angular velocity (wearable sensors).

Alzheimer’s pathologies were not significantly associated with any parameters of the 360° turn test as a single task when adding adjusting factors, see Table 2 and STable 3.

In contrast, WMH remained significantly associated with increased turn duration during the 360° turn as a single task, that is, after adjusting for age, sex, ICV, stroke/TIA, and education. This applied for stopwatch data (B 0.04, 95% CI 0.01, 0.08; *p *= .018) as well as sensor data (B 0.05, 95% CI 0.01, 0.10; *p* = .016), see Table 2.

### Turning 360° in standing and dual-tasking: associations with brain pathologies

After adjustment for age, sex, ICV, stroke/TIA, and education, WMH were no longer significantly associated (*p* ≥ .073) with any of the turning parameters during dual-tasking (360°turn), see Table 2. In contrast, Alzheimer’s pathologies remained significantly associated with being unstable while dual-tasking, see Table 2. This applied for Aβ (OR 10.08, 95% CI, 1.30, 77.70; *p* = .027) as well as tau (OR 37.24, 95% CI, 2.64, 523.79; *p* = .007), see Table 2.

In a subsequent step, both Aβ and tau were simultaneously entered in the same model while adjusting for age, sex, stroke/TIA, and education. Aβ was then no longer significant (*p* = .164), whereas tau remained significantly associated with being unstable during dual-tasking: OR 22.42, 95% CI, 1.43, 349.88; *p* = .027, see Table 4. Tau remained significantly associated in sensitivity analyses, see Table 4, including its footnotes.

Additional sensitivity analyses included subgroups who were without fear of falling and/or without a history of falls, see STable 6A-C. Among those without fear of falling, amyloid pathology (Aβ) was independently associated with being unstable during dual-tasking (360° turn). It remained significantly associated if also excluding those with a history of falls.

### Turning 180° while walking (sensor: peak angular velocity and turn duration): associations with brain pathologies

Alzheimer’s pathologies (ie, Aβ and tau) showed no associations (*p* ≥ .417) with any of the parameters in relation to a 180° turn (ie, as a single task or while dual-tasking), see Table 3, STables 4 and 5.

In analyses that included adjusting factors, WMH were associated with a decreased peak angular velocity: B −1.27 (95% CI −2.33, −0.21, *p* = 0.018), see Table 3.

## Discussion

This study contributes new knowledge of how Alzheimer’s and vascular pathologies are associated with turning performance (during single and dual tasks) in cognitively healthy older people. Although both Alzheimer’s and vascular brain changes seem to be associated with the challenging motor task of turning, the associations seem to differ depending on the type of turn and whether it is assessed as a single- or dual-task. Our main findings suggest that early Alzheimer’s pathologies are associated with instability while turning 360° and dual-tasking. In contrast, vascular pathologies (WMH) seem to be associated with turning as a single task, that is, an increased duration (360° turn) and a decreased peak angular velocity (180° turn). Importantly, our findings apply to older people without cognitive impairments, that is, “pre-symptomatic”. These findings support further studies of early motor involvement in neurodegenerative diseases, where turning seems a promising motor task to address. Moreover, a 360° turn seems important to consider when using dual-task assessments. Longitudinal studies are needed to establish whether turning performance can be of prognostic value for future cognitive decline.

This study specifically focused on Alzheimer’s pathologies. When adjusting for covariates, increased levels of Aβ and tau were only independently associated with instability during turning 360° and dual-tasking—that is, not when turning as a single task, see Table 2. This corroborates the utility of using dual task assessments to detect subtle motor impairments. Moreover, turning 360° seems promising to use as the motor task in dual-task assessments.^17^ Benefits of the assessment of instability in this test include that it does not require technical equipment, needs limited space, and takes little time to administer. That is, it is easily implemented in clinical practice.

In analyses limited to one brain pathology, elevated Aβ levels were significantly associated with instability during dual-tasking (360° turn) when including adjusting factors, see Table 2. This aligns with prior studies showing associations between Aβ and reduced balance control and falls.^24–27^ However, the association with Aβ did not persist when also including tau in the same model, see Table 4. On the other hand, increased levels of tau remained independently associated with being unstable while dual-tasking and turning 360°, which also persisted in sensitivity analyses (see Table 4 and footnotes). Importantly, tau pathology is more associated with cognitive impairment than Aβ pathology.^45^^,^^46^ The current finding aligns with some CSF studies that demonstrated an association between tau and decreased dual-task motor performance in mixed samples, which included cognitively healthy controls.^27^^,^^31^^,^^47^ Only one of these studies considered simultaneously both Aβ and tau.^27^ Another two CSF studies reported conflicting findings regarding the association between tau and mobility while dual-tasking, but these did not include cognitively healthy participants.^34^^,^^48^ None of the former studies used PET imaging, nor did they address turning.^27^^,^^31^^,^^34^^,^^47^^,^^48^

Our novel finding in relation to turning and dual-tasking is of specific interest as our sample was cognitively healthy; it indicates that being unstable while turning and dual-tasking may be an early sign for those at risk of future cognitive decline. Our findings corroborate that dual-tasking can negatively affect balance, especially if the motor task is challenging.^49^

It should be noted that Alzheimer’s pathology showed an association with instability while dual tasking, also in a subgroup without fear of falling, and without a history of falls. These findings suggest that cognitively healthy older people with Alzheimer’s pathologies should be recommended balance training, that is, to improve or maintain balance control while dual-tasking.

In contrast with our findings in relation to turning 360°, Alzheimer’s pathologies showed no significant association with turning 180° when adding adjusting factors, that is, as a single task or while dual-tasking. This discrepancy may reflect the absence of an instability measure in relation to the 180° turn. Another potential explanation is that turning 360° in standing (both directions) is more challenging than turning 180° while walking; a 360° turn may therefore be more sensitive for early Alzheimer’s pathologies. This aligns with prior studies, which suggested that smaller turns (ie, fewer degrees of the turn) are less demanding than larger turns.^8^^,^^10^ Moreover, in the current study, the complexity of turning 360° while dual-tasking is supported both by the number of participants who required a second trial (87 participants) and by the fact that 14 participants did not manage the test at all. In comparison, only two participants did not manage the TUG while dual-tasking. Moreover, the findings may reflect differences in the time duration of the two tests. That is, a test with a short duration (ie, a 180° turn) may not be as effective in capturing cognitive-motor interference (dual task effect) in cognitively healthy older people. One may therefore wonder why the two 180° turns in TUG were not merged. The reasoning was that these two turns differ in their execution. The first turn in TUG relates purely to walking. The second turn is initiated while walking, but it ends with a stop before sitting down. Although the current study showed that Alzheimer’s pathologies were not associated with the performance of a 180° turn in cognitively healthy older people, a prior study reported that the velocity of a 180° turn while dual tasking differentiated between three groups (dementia, *n* = 42; MCI, *n* = 42; normal cognitive function, *n* = 38).^50^ Longitudinal studies that address different types of turns are required to investigate whether, and which, turning parameters may be of prognostic value for future cognitive decline and different diagnoses.

This study showed that increased levels of WMH were associated with an increased duration of the 360° turn test when adjusting for age, sex, stroke/TIA, education, and ICV. However, this was only the case for turning in the single task situation. This also applied to the 180° turn, where increased levels of WMH were associated with a decreased peak angular velocity, but only in the single task situation. Although this may be somewhat surprising, it corroborates our previous study (based on another sample) that addressed mobility among people without dementia.^27^ In the total sample, WMH were associated with mobility as a single task, but not while dual-tasking. However, sub-analyses showed that this applied for people with MCI, but not for cognitively unimpaired, where WMH showed no association with mobility (as a single task or while dual-tasking). The latter contradicts our findings, which may reflect sample size differences (145 versus 297 in the current study) and/or differences in the motor task (mobility versus turning). The former study used a similar subtraction task (-3). Prior studies showed inconsistent findings regarding the associations between dual-task walking and cerebral small vessel disease.^2^ It should be noted that the amount of WMH was relatively small (Table 1) in the current study, that is, as suspected in a cognitively healthy cohort.

Turning performance is associated with cognitive functioning among older people without dementia.^7^^,^^9^^,^^13^^,^^14^ Moreover, turning is a complex motor activity that requires sensory-motor integration. Several networks in the brain seem to be involved, for example, the salience-, the cingulo-opercular-, and the dorsal attention network.^51^ The latter is negatively affected already in preclinical Alzheimer’s disease.^52^ In addition, dual-task performance seems to involve the premotor cortex, frontal operculum (important for complex tasks), anterior intraparietal sulcus (pivotal for multisensory integration), the left inferior frontal gyrus (important for executive functioning), and the left inferior frontal sulcus.^53^ The default mode network (DMN) seems also important for dual-task performance.^54^ In DMN, disease-specific pathology occurs already in preclinical Alzheimer’s disease, which affects brain connectivity.^55^ Several of these regions and networks also play a role in balance control, which might reflect our finding that Alzheimer’s pathology was independently associated with instability while dual tasking and turning.

Our findings imply that Alzheimer’s pathology is independently associated with decreased balance while turning and dual tasking. This also indicates that the motor task was not prioritized while dual-tasking, which affected balance. These findings signal that dual task-assessments (turning 360°+ subtraction task) seem especially sensitive to early Alzheimer’s pathologies. On the other hand, if you are interested in early markers of vascular dementia, our results suggest that WMH were independently associated with the duration of 360° turns, and peak angular velocity of a 180° turn, that is, as single tasks. That is, early vascular changes seem to impact temporal features of turning and not instability while turning 360°. The lack of association between WMH and turns while dual tasking is difficult to grasp. Still, prior studies showed inconsistent findings regarding the associations between dual-task walking and cerebral small vessel disease.^2^ The current finding might reflect that the participants prioritized the motor task while dual-tasking. On a group level, both the turning and the cognitive task deteriorated while dual tasking (indicating mutual interference). Future studies should explicitly investigate task prioritization, which requires that several strategies of cognitive-motor interference are addressed.^56^

All considered, the findings imply that early brain pathologies seem to affect turns and turning parameters differently. To gain a deeper understanding, we need longitudinal studies that address brain pathologies and several types of turns and turning parameters, that is, both as single tasks and while dual tasking. Still, turning assessments seem promising for investigating motor aspects in very early stages of Alzheimer’s disease and vascular brain disease.

The external validity of our findings applies to cognitively healthy older people, and the results of dual-tasking refer to turns as the motor task and a cognitive subtraction task.

### Methodological considerations (strengths and limitations)

This study has several strengths, such as the inclusion of a well-characterized cohort, the use of advanced imaging (tau and amyloid PET as well as MRI), and that we included different turns (a 360° turn in standing and a 180° turn while walking), as well as including both clinical and digital assessments. Moreover, turning was assessed both as a single task and while dual-tasking, which was also the case for the cognitive task. Another strength is the use of similar subtraction tasks (-3) to facilitate comparisons between the two turning tasks while dual-tasking. One could argue that a subtraction task by 7 would have challenged turning performance even more, but our choice was based on the short duration of a turn, that is, as compared to gait protocols that last longer.

This study also has limitations. The digital assessments were implemented later, which yielded smaller sample sizes in these comparisons. We included 297 participants, of whom 180 had sensor data. However, a recent scoping review (targeted instrumented analyses of 360-degree turn) reported that only two out of 21 studies had a sample size that exceeded 100 participants. Moreover, most studies concerned people with Parkinson’s disease.^57^

In addition, using wearable sensors in the home setting could have rendered more ecologically valid data. However, this cohort will be followed for eight years, and the environment was therefore chosen as a fixed factor.

It should be noted that several additional diagnoses (eg, osteoarthritis) and additional variables may affect turning parameters, such as pain and lower extremity strength.

In addition, the current cross-sectional design can be considered a weakness, which should be kept in mind when interpreting the results. The latter also applies to the use of multiple comparisons.

Finally, our findings apply to cognitively healthy older people and should be replicated in similar cohorts in the future. The external validity for other cohorts remains unknown. Still, addressing cognitively healthy people is a strength, that is, if interested in early motor signs of those at risk of developing cognitive decline. This suggests that brain pathologies precede a future cognitive decline.

## Conclusions

This study suggests that Alzheimer’s pathologies are independently associated with instability while turning 360° and dual-tasking in cognitively healthy older people, while vascular pathology seems to be associated with turning as a single task. Our cross-sectional findings should be verified in additional samples of cognitively healthy older people and in longitudinal studies. Still, turning assessments seem promising for investigating motor aspects in very early stages of Alzheimer’s disease and vascular brain disease. More specifically, our findings indicate that assessing instability while turning 360° and dual-tasking seems promising for investigating motor aspects in very early stages of Alzheimer’s disease.

## Supplementary material

Supplementary material is available at *The Journals of Gerontology, Series A: Biological Sciences and Medical Sciences* online.

## Supplementary Material

glag114_Supplementary_Data

## Data Availability

All descriptive data generated or analyzed during this study are included in this published article, that is, on a group level. The original datasets used and/or analyzed during the current study are available from the corresponding author on reasonable request. The latter implies that pseudonymized data can be shared by request from qualified academic investigators for the sole purpose of replicating procedures and results presented in the article, that is, if the data transfer agrees with both EU legislation on the general data protection regulation and decisions by the Swedish Ethical Review Authority and Region Skåne, which should be regulated in a material transfer agreement.
