# Supplementary material for: Turning as a single and dual task—associations with Alzheimer’s and vascular pathologies in cognitively healthy older people
Source: J Gerontol A Biol Sci Med Sci. 2026 May 7;81(7):glag114. doi: 10.1093/gerona/glag114 (PMC13274543; doi:10.1093/gerona/glag114)
Supplement: glag114_Supplementary_Data [file glag114_supplementary_data.docx]

Title: Turning as a Single and Dual Task - Associations With Alzheimer’s and Vascular Pathologies in Cognitively Healthy Older People

Supplementary material

1. **SMethods**
2. **STable 1.** The cognitive task as a single task and while dual-tasking, including the motor tasks for comparisons.
3. **STable 2**. Simple regression (linear or logistic) analyses (unadjusted) with different turning 360° parameters as dependent variables in cognitively healthy older people.
4. **STable 3**. Multivariable regression analyses (linear or logistic, each pathology was adjusted for age and sex) with different turning 360° parameters as dependent variables in cognitively healthy older people.
5. **STable 4.** Simple regression linear regression analyses (unadjusted) with different turning 180° parameters as dependent variables in cognitively healthy older people.
6. **STable 5.** Multivariable linear regression analyses (each pathology was adjusted for age and sex) with different turning 180° parameters as dependent variables in cognitively healthy older people.
7. **STable 6.** Sensitivity analyses in three sub-groups: A) those without fear of falling, B) those without a history of falls, and C) those without fear of falling and a history of falls.

**S. Methods**

**Recruitment**

Community dwelling older adults were recruited by using random sampling from two prior population-based community cohort studies: the Malmö Diet and Cancer Study (i.e., those participating in the early 1990’s) (1, 2), and a minority was recruited from the Malmö Offspring Study.

*References that describe the Malmö Diet and Cancer study*

1) Manjer J, Carlsson S, Elmståhl S, et al. The Malmö Diet and Cancer Study: representativity, cancer incidence and mortality in participants and non-participants. *Eur J Cancer Prev*. Dec 2001;10(6):489–99. doi:10.1097/00008469-200112000-00003

2) Manjer J, Elmståhl S, Janzon L, Berglund G. Invitation to a population-based cohort study: differences between subjects recruited using various strategies. *Scand J Public Health*. 2002;30(2):103–12. doi:10.1177/14034948020300020401

**Cognitive assessments**

- Global cognition was assessed by using the Mini-Mental State Examination (MMSE).

# Memory was assessed using the 10-word delayed recall test from the Alzheimer’s Disease Assessment Scale–Cognitive Subscale (ADAS-Cog).

- Verbal ability was assessed using animal fluency and the 15-item short version of the Boston Naming Test.
- Attention and executive function were assessed by using the Trail Making Test (TMT) A and B, and the Symbol Digit Modalities Test (SDMT).
- Visuospatial ability was measured by using incomplete letters and cube analysis from the Visual Objects and Space Perception (VOSP) battery.

A modified Preclinical Alzheimer’s Cognitive Composite (mPACC5) was calculated as follows: ([ADAS delayed recall x 2] + animal fluency + MMSE + SDMT) / 5

Delayed recall was given a double weight to maintain the same proportion of memory as in the original composite score. All cognitive test scores were initially transformed into z-scores, i.e., prior to the mPACC5 calculation.

The detailed cognitive assessments preceded turning assessments. The median (q1-q3) delay was 20 days (9-36); 54 participants completed these assessments on the same day.

**The cognitive subtraction task (minus 3) in relation to dual-task assessments**

- In the single task setting, the participant was asked to do serial subtractions by 3 (starting from 30), which was done in a sitting position. All participants were given 10 seconds to complete the task. If a participant completed the task (i.e., reaching zero) faster than 10 seconds, the faster time was registered. The following was also registered: the total number of stated answers (i.e., total number of subtractions), and the number of correct subtractions.
- In the dual task setting, the participant was asked to subtract 3 starting from 100 while simultaneously performing the Timed up & Go test at a comfortable pace. The participant started from 99 while simultaneously performing the 360° turn test. No instructions regarding prioritization were given. The following was registered: the total number of subtractions, and the number of correct subtractions. The time for completing the motor task was also registered.

*Clarifications regarding measures while dual tasking*

In relation to the 360° turn, we have exact time measures that relate to the cognitive task while dual tasking. However, this is not the case for the 180° turn while walking, i.e., as the cognitive subtraction task was done throughout the Timed Up and Go test. In the latter case, accuracy and speed measures of the subtraction task therefore relate to the performance of the Timed Up and Go Test.

*Calculations (accuracy and speed), as a single task and while dual-tasking*

Accuracy of the cognitive tasks was calculated as: the number of correct subtractions / the total number of subtractions. This ratio was then multiplied by 100 to represent a percentage.

The speed of the performance of the cognitive task was calculated in two different ways.

1. Total number of completed subtractions per second

The total number of completed subtractions was divided with the time taken to complete the task.

In the single task setting, if the task was completed faster than 10 seconds, the value was divided by the registered time.

In the dual task setting, the value was divided by the time it took to complete the motor task (i.e., Timed up & Go and the 360° turn test, respectively).

1. The number of correct subtractions per second

The number of correct subtractions was divided with the time taken to complete the task.

In the single task setting, the value (number of correct subtractions) was divided by the time it took to complete the task. If the task was completed faster than 10 seconds, then the value was divided by the registered time.

In the dual task setting, the value was divided by the time it took to complete the motor task (i.e., Timed up & Go and the 360° turn test, respectively).

*Reference for calculations of speed and accuracy*

Kim et al. Dual Task Effects on Speed and Accuracy During Cognitive and Upper Limb Motor Tasks in Adults with Stroke Hemiparesis. Front Hum Neurosci 2021; 17:15:671541.doi: 10.3389/fnhum.2021.671541. eCollection 2021.

**Imaging**

The three imaging scans were performed within 6 months for all except five individuals. One of the five had a little bit more than 9 months between MRI and tau-PET (273 days) and 8.5 months (258) days between MRI and Aβ PET. A second person had roughly 7 months (221 days) between MRI and Aβ-PET scan. A third person had the tau-PET scan performed around 7 months (212 days) from MRI. A fourth person had a difference of 7 months (215 days) between PET scans. The fifth person had a difference of 6 months and 9 days (i.e., 189 days) between the PET scans. For the four latter individuals, the difference can be explained by severe restrictions due to the covid pandemic in Sweden 2020-2021. However, most of the sample had their three scans within a rather short period.

The median (q1-q3) delay between turning assessments and MRI was 15 days (6.8-30). For Aβ PET, the median delay was 37 days (20–56.8), and for tau PET, 22 days (9–46).

The mean number of days between the scans were as follows:

- MRI and tau PET: median (q1-q3) 18 days (6 - 45)
- MRI and Aβ PET: median (q1-q3) 32 days (19 - 59)
- Tau-PET and Aβ PET: median (q1-q3) 29 days (13 - 48.5)

| Cognitive variables (subtraction task) | Single^a^ | Dual^b^ | Sign. | Missing, n  Single/dual |
| --- | --- | --- | --- | --- |
|  |  |  |  |  |
| Accuracy^1^ (%), median (q1 - q3) | 100^2^ (100 - 100) | 100 (89 - 100)^3^ | **p< 0.001^4^** | 3 / 2 |
| Speed. Correct subtractions per second, mean (SD) | 0.62 (0.30) | 0.44 (0.18) | **p< 0.001^5^** | 3 / 2 |
| Speed. Total number of subtractions per second, mean (SD) | 0.63 (0.29) | 0.46 (0.17) | **p< 0.001^5^** | 3 / 2 |
| Motor variables  Timed Up & Go, Turning 180° | Single, | Dual | Sign. |  |
|  |  |  |  |  |
| Timed up & Go, total time, stopwatch (s), mean (SD) | 10.3 (2.1) | 13.7 (4.3) | **p< 0.001^5^** | - / 2 |
|  |  |  |  |  |
| Turning 180° (sensor data), n= 180 |  |  |  |  |
| Peak angular velocity (°/s), mean (SD) | 141.3 (31.7) | 120.0 (29.2) | **p< 0.001^5^** | - / 2 |
| Turn duration (s), mean (SD) | 3.0 (0.5) | 3.3 (0.6) | **p< 0.001^5^** | - / 2 |

**STable 1A.** Cognitive subtraction tasks and motor variables that relate to a 180° turn, single and dual tasking, n = 297.

^1^ Formula: correctly stated numbers/ stated numbers. The value is then multiplied by 100 to present a percentage. ^2^ 265 individuals answered all stated numbers correctly. ^3^ 217 individuals answered all stated numbers correctly. ^4^ Wilcoxon signed rank test was used. ^5^ Paired samples t-test was used.

^a^The cognitive single task is performed in a seated position. The participant is asked to subtract 3 starting from 30, during 10 seconds. If the participants reaches zero before the 10 seconds have passed, the time taken to complete the task is registered and used.

^b^The dual task was performed as followed: The participant is instructed to perform the Timed up & Go test at a comfortable pace. The participant is asked to simultaneously perform a subtraction task (-3 starting from 100). No instruction regarding prioritization of task is given.

Bolded p-values are statistically significant. Please see **SMethods** for further clarifications.

| Cognitive variables (subtraction task) | Single^a^ | Dual^b^ | Sign. | Missing, n  Single/dual |
| --- | --- | --- | --- | --- |
|  |  |  |  |  |
| Accuracy^1^ (%) median (q1 - q3) | 100^2^ (100 - 100) | 100 (83 - 100) | **p< 0.001^3^** | 3 / 19 |
| Speed. Correct subtractions per second, mean (SD) | 0.62 (0.30) | 0.45 (0.21) | **p< 0.001^4^** | 3 / 19 |
| Speed. Total number of subtractions per second, mean (SD) | 0.63 (0.29) | 0.50 (0.19) | **p< 0.001^4^** | 3 / 19 |
| Motor variables  Turning 360° in standing (both directions) | Single, | Dual | Sign. |  |
|  |  |  |  |  |
| Clinical assessments, n= 297 |  |  |  |  |
| Unstable (= scored 0-3), n (%) | 114 (38.8) | 137 (48.9) | **p=0.004^5^** | 3 / 17 |
| Timed, both turns, stopwatch (s), mean (SD) | 6.7 (1.5) | 9.8 (3.4) | **p< 0.001^6^** | 3 / 17 |
| Sensor variables, n= 180 |  |  |  |  |
| Peak angular velocity (°/s), mean (SD) | 207.9 (44.5) | 167.2 (41.4) | **p< 0.001^7^** | 2 / 11 |
| Turn duration (s), mean (SD) | 6.5 (1.3) | 9.2 (2.4) | **p< 0.001^7^** | 2 / 11 |

**STable 1B.** Cognitive subtraction tasks and motor variables that relate to a 360° turn, single and dual tasking, n = 297.

^1^ formula: correctly stated numbers/ stated numbers. The value is then multiplied by 100 to present a percentage. ^2^ 265 individuals answered all stated numbers correctly. ^3^ Wilcoxon signed rank tests were used. N=276. ^4^ Paired samples t-test was used. N=276. ^5^ McNemar test was used. N=280. ^6^ Paired samples t-test was used. N=280. ^7^ Paired samples t-test was used. N=169.

^a^The cognitive single task is performed in a seated position. The participant is asked to subtract 3 starting from 30, during 10 seconds. If the participants reaches zero before the 10 seconds have passed, the time taken to complete the task is annotated.

^b^The dual task was performed as follows: The participant is instructed to perform a 360° turn to one side, stop briefly, and then turn 360° to the other side. The participant is asked to simultaneously perform a subtraction task (-3 starting from 9). No instruction was given regarding the prioritization of tasks.

Bolded p-values are statistically significant.

**STable 2. Simple regression (linear or logistic) analyses (unadjusted) with different turning 360° parameters as dependent variables in cognitively healthy older people.**

|  | **Turning 360° in standing- single task** | | | |
| --- | --- | --- | --- | --- |
|  | Timed task, stopwatch (s)  n=294 | Unstable (coded 1)  n=294 | Peak angular velocity, sensor (degrees / s)  n=178 | Turn duration, sensor (s)  n=178 |
|  | B (95 % CI)  p-value | OR (95 % CI)  p-value | B (95 % CI)  p-value | B (95 % CI)  p-value |
| Aβ PET, SUVR | 0.51 (-0.77, 1.80)  p=0.431 | 5.41 (0.94, 30.92)  p=0.058 | -1.76 (-48.24, 44.73) p=0.941 | 0.41 (-0.96, 1.77) p=0.557 |
| Tau PET, SUVR | 1.50 (0.09, 2.91) **p=0.037** | 5.36 (0.69, 41.36)  p=0.107 | -2.16 (-71.65, 67.33) p=0.951 | -0.22 (-2.26, 1.81) p=0.830 |
| WMH, mL | 0.05 (0.02, 0.09) **p<0.001** | 1.04 (0.99, 1.09) p=0.112 | -1.82 (-3.22, -0.43) **p=0.011** | 0.08 (0.03, 0.12) **p<0.001** |
|  | **Turning 360° in standing- dual tasking** | | | |
|  | Timed task, stopwatch (s)  n=280 | Unstable (coded 1)  n=280 | Peak angular velocity, sensor (degrees / s)  n=169 | Turn duration, sensor (s)  n=169 |
| Aβ PET, SUVR | 3.02 (-0.06, 6.10) p=0.054 | 24.14 (3.33, 174.87)  **p=0.002** | -9.63 (-54.33, 35.08)  p=0.671 | 1.54 (-1.09, 4.17)  p=0.248 |
| Tau PET, SUVR | 2.73 (-0.63, 6.08)  p=0.111 | 31.27 (2.58, 378.55)  **p=0.007** | -5.12 (-72.15, 61.91) p=0.880 | 1.84 (-2.10, 5.79) p=0.357 |
| WMH, mL | 0.08 (0.01, 0.15) **p=0.043** | 1.07 (1.01, 1.12) **p=0.009** | -1.22 (-2.52, 0.07) p=0.063 | 0.09 (0.01, 0.18) **p=0.021** |

*Note.* In models that included WMH, 3-4 had missing data. B = unstandardized regression coefficient; CI = confidence interval; OR = odds ratio; Aβ = amyloid-β; PET = positron emission tomography; SUVR = standardized uptake value ratio; WMH = white matter hyperintensities (assessed by using magnetic resonance imaging).

Aβ PET SUVR was according to a neocortical meta region of interest (ROI): prefrontal, lateral temporal, parietal, anterior cingulate and posterior cingulate/precuneus, using pons as the reference region. A composite temporal meta ROI was used for tau pathology (i.e., entorhinal cortex, inferior and middle temporal cortices, fusiform gyrus, parahippocampal cortex and amygdala), using the inferior cerebellar cortex as reference region. The stability variable (original scoring 0-4, higher = better) was dichotomized as follows: unstable (scores 0-3, coded as 1) and good balance/stable (score 4, coded as 0). Bolded p-values are statistically significant.

**STable 3. Multivariable regression analyses (linear or logistic, each pathology was adjusted for age and sex) with different turning 360° parameters as dependent variables in cognitively healthy older people.**

|  | **Turning 360° in standing- single task** | | | |
| --- | --- | --- | --- | --- |
|  | Timed task, stopwatch (s)  n=294 | Unstable (coded 1)  n=294 | Peak angular velocity, sensor (degrees / s)  n=178 | Turn duration, sensor (s)  n=178 |
|  | B (95 % CI)  p-value | OR (95 % CI)  p-value | B (95 % CI)  p-value | B (95 % CI)  p-value |
| Aβ PET, SUVR | -0.13 (-1.40, 1.14) p=0.836 | 2.59 (0.42, 15.86) p=0.305 | 12.70 (-34.01, 59,40) p=0.592 | -0.22 (-1.53, 1.10) p=0.746 |
| Tau PET, SUVR | 1.13 (-0.24, 2.50) p=0.104 | 4.08 (0.50, 32.95) p=0.187 | -6.55 (-75.14, 62.03) p=0.851 | -0.06 (-1.99, 1.87) p=0.951 |
| WMH, mL | 0.04 (0.01, 0.08) **p=0.013** | 1.01 (0.96, 1.06)  p=0.658 | -1.48 (-2.98, 0.02) p=0.052 | 0.06 (0.01, 0.10) **p=0.008** |
|  | **Turning 360° in standing- dual tasking** | | | |
|  | Timed task, stopwatch (s)  n=280 | Unstable (coded 1)  n=280 | Peak angular velocity, sensor (degrees / s)  n=169 | Turn duration, sensor (s)  n=169 |
| Aβ PET, SUVR | 2.05 (-0.97, 5.06) p=0.183 | 11.41 (1.50, 86.65) **p=0.019** | -3.78 (-48.89, 41.33) p=0.869 | 1.32 (-1.24, 3.87) p=0.310 |
| Tau PET, SUVR | 1.79 (-1.42, 5.00) p=0.273 | 33.66 (2.52, 448.28) **p=0.008** | -4.09 (-70.22, 62.04) p=0.903 | 1.58 (-2.17, 5.32) p=0.407 |
| WMH, mL | 0.05 (-0.03, 0.13) p=0.209 | 1.05 (0.99, 1.10)  p=0.079 | -0.95 (-2.36, 0.45) p=0.181 | 0.07 (-0.02, 0.16) p=0.100 |

*Note.* Models including WMH were also adjusted for intracranial volume. In models that included WMH, 3-4 had missing data. B = unstandardized regression coefficient; CI = confidence interval; OR = odds ratio; Aβ = amyloid-β; PET = positron emission tomography; SUVR = standardized uptake value ratio; WMH= white matter hyperintensities (assessed by using magnetic resonance imaging).

Aβ PET SUVR was according to a neocortical meta region of interest (ROI): prefrontal, lateral temporal, parietal, anterior cingulate and posterior cingulate/precuneus, using pons as the reference region. A composite temporal meta ROI was used for tau pathology (i.e., entorhinal cortex, inferior and middle temporal cortices, fusiform gyrus, parahippocampal cortex and amygdala), using the inferior cerebellar cortex as reference region. The stability variable (original scoring 0-4, higher = better) was dichotomized as follows: unstable (scores 0-3, coded as 1) and good balance/stable (score 4, coded as 0). Bolded p-values are statistically significant.

**STable 4. Simple regression linear regression analyses (unadjusted) with different turning 180° parameters as dependent variables in cognitively healthy older people.**

|  | **Turning 180° while walking (sensor)** | | | |
| --- | --- | --- | --- | --- |
|  | Single task | Dual tasking | Single task | Dual tasking |
|  | Peak angular velocity (degrees / s)  n=180 | Peak angular velocity (degrees / s)  n=178 | Turn duration (s)  n=180 | Turn duration (s)  n=178 |
|  | B (95 % CI)  p-value | B (95 % CI)  p-value | B (95 % CI)  p-value | B (95 % CI)  p-value |
| Aβ PET, SUVR | -7.76 (-40.75, 25.24) p=0.643 | -11.24 (-41.60, 19.12) p=0.466 | 0.10 (-0.47, 0.67) p=0.724 | 0.21 (-0.43, 0.85) p=0.518 |
| Tau PET, SUVR | 19.52 (-29.63, 68.68) p=0.434 | -10.54 (-55.88, 34.80**)** p=0.647 | -0.02 (-0.87, 0.83) p=0.959 | 0.11 (-0.84, 1.07) p=0.814 |
| WMH, mL | -1.51 (-2.50, -0.52) **p=0.003** | -0.82 (-1.73, 0.09) p=0.075 | 0.02 (0.01, 0.05) **p=0.005** | 0.00 (-0.02, 0.03) p=0.666 |

*Note.* In models that included WMH, 3 had missing data. B = unstandardized regression coefficient; CI = confidence interval; Aβ = amyloid-β; SUVR = standardized uptake value ratio; PET = positron emission tomography; WMH = white matter hyperintensities (assessed by using magnetic resonance imaging).

Aβ PET SUVR was according to a neocortical meta region of interest (ROI): prefrontal, lateral temporal, parietal, anterior cingulate and posterior cingulate/precuneus, using pons as the reference region. A composite temporal meta ROI was used for tau pathology (i.e., entorhinal cortex, inferior and middle temporal cortices, fusiform gyrus, parahippocampal cortex and amygdala), using the inferior cerebellar cortex as reference. region. Bolded p-values are statistically significant.

**STable 5. Multivariable linear regression analyses (each pathology was adjusted for age and sex) with different turning 180° parameters as dependent variables in cognitively healthy older people.**

|  | **Turning 180° while walking (sensor)** | | | |
| --- | --- | --- | --- | --- |
|  | Single task | Dual tasking | Single task | Dual tasking |
|  | Peak angular velocity (degrees / s)  n=180 | Peak angular velocity (degrees / s)  n=178 | Turn duration (s)  n=180 | Turn duration (s)  n=178 |
|  | B (95 % CI)  p-value | B (95 % CI)  p-value | B (95 % CI)  p-value | B (95 % CI)  p-value |
| Aβ PET, SUVR | 2.39 (-30.55, 35.32) p=0.887 | -7.24 (-38.04, 23.57) p=0.643 | -0.04 (-0.62, 0.54) p=0.892 | 0.17 (-0.48, 0.82) p=0.601 |
| Tau PET, SUVR | 18.59 (-29.52, 66.71) p=0.447 | -9.22 (-54.30, 35.86) p=0.687 | 0.01 (-0.83, 0.85) p=0.984 | -0.03 (-0.92, 0.97) p=0.957 |
| WMH, mL | -1.34 (-2.39, -0.30) **p=0.012** | -0.78 (-1.75, 0.20) p=0.116 | 0.02 (0.00, 0.04) p=0.054 | 0.00 (-0.02, 0.03) p=0.723 |

*Note.* Models including WMH were also adjusted for intracranial volume; these models had three missing values. B = unstandardized regression coefficient; CI = confidence interval; Aβ = amyloid-β; SUVR = standardized uptake value ratio; PET = positron emission tomography; WMH = white matter hyperintensities (assessed by using magnetic resonance imaging).

Aβ PET SUVR was according to a neocortical meta region of interest (ROI): prefrontal, lateral temporal, parietal, anterior cingulate and posterior cingulate/precuneus, using pons as the reference region. A composite temporal meta ROI was used for tau pathology (i.e., entorhinal cortex, inferior and middle temporal cortices, fusiform gyrus, parahippocampal cortex and amygdala), using the inferior cerebellar cortex as reference region. Bolded p-values are statistically significant.

**STable 6. Sensitivity analyses. Logistic regression analyses in subgroups of the total sample. Dependent variable: unstable while dual tasking (turn 360°)**

These analyses concern three subgroups: those without fear of falling, those who reported no falls in the past 12 months, those without fear of falling and without a history of falls (past 12 months). The results are presented in three tables:

- STable 6A includes those without fear of falling.
- STable 6B includes those without a history of falls (past 12 months).
- STable 6C includes those without fear of falling and without a history of falls (past 12 months).

**General information regarding all three tables**

- The dependent variable in all three tables: Instability while turning 360° and dual tasking.

The dependent variable (original scoring 0-4, higher = better) was dichotomized as follows: unstable (scores 0-3, coded as 1) and good balance/stable (score 4, coded as 0).

- Each regression model included both Aβ and tau, and all models were adjusted for age, sex, stroke/TIA and education.
- Aβ PET SUVR was according to a neocortical meta region of interest (ROI): prefrontal, lateral temporal, parietal, anterior cingulate and posterior cingulate/precuneus, using pons as the reference region.
- A composite temporal meta ROI was used for tau pathology (i.e., entorhinal cortex, inferior and middle temporal cortices, fusiform gyrus, parahippocampal cortex and amygdala), using the inferior cerebellar cortex as reference region.
- Cognition: a modified Preclinical Alzheimer’s Cognitive Composite (mPACC5) was calculated as follows: ([ADAS delayed recall x 2] + animal fluency + MMSE + SDMT) / 5.

**General information and comments about the total sample and sub-groups**

- The total sample was cognitively healthy older people. This selection does in itself have an impact on the participants’ motor features as shown in Table 1. For example, our sample has a high gait speed, and a low proportion of fallers in relation to their mean age. This is not a surprising finding as cognitive and motor functions are related. Excluding those with falls and/or fear of falling implies an even healthier cohort.
- In the total sample, two needed mobility devices, 14 did not manage the test, and 1 had missing data. That is, 17 participants were excluded or had missing data. Seven of those reported falls, five reported fear of falling, and two reported both falls and fear of falling.

The above resulted in a sample of 280 participants. Sixty-seven (24%) of those reported falls, 79 (28%) reported fear of falling, and 25 (9%) reported both falls and fear of falling. This reflects that a history of falls and fear of falling are not interchangeable constructs.

**STable 6A.** Multivariable logistic regression analysis with instability during 360° turning and dual-tasking as the dependent variable in cognitively healthy older adults **without fear of falling**.

Each model included both Aβ and tau, and all models were adjusted for age, sex, stroke/TIA and education.

|  | Turning 360° in standing- being unstable while dual tasking (coded 1) | | |
| --- | --- | --- | --- |
|  | Original model | Sensitivity analysis  (excl. 2 outliers tau-PET) ^1^ | Sensitivity analysis  (excl. 2 outliers tau-PET + adjusting for cognition) ^2, 3^ |
|  | n=197 | n=195 | n=195 |
|  | OR (95 % CI)  p-value | OR (95 % CI)  p-value | OR (95 % CI)  p-value |
| Aβ PET, SUVR | 36.26 (2.12, 620.57)  **p=0.013** | 35.84 (2.10, 610.47)  **p=0.013** | 38.95 (2.23, 678.21)  **p=0.012** |
| Tau PET, SUVR | 14.26 (0.49, 418.80) p=0.123 | 12.26 (0.34, 431.61) p=0.168 | 13.22 (0.0,36, 474.93)  p=0.158 |

*Note.* TIA = transient ischemic attack; OR = odds ratio; CI = confidence interval; Aβ = amyloid-β; PET = positron emission tomography;

SUVR = standardized uptake value ratio. Bolded p-values are statistically significant.

In this subsample, 91out of 197 (46.2%) were unstable while dual-tasking, and 42 (21.3 %) reported a history of falls.

^1^ If also adjusting for cardiac history, the results were as follows. Aβ PET 29.97 (1.74, 515.52), **p=0.019,**

Tau PET: 13.64 (0.37, 491.38), p=0.153, n=195.

^2^ If adjusting for comfortable gait speed instead of cognition, the results were as follows. Aβ PET: 37.41 (2.18, 641.94), **p=0.013**,

Tau PET: 13.82 (0.36, 525.52), p=0.157. n= 192.

Aβ remained significant if also adjusting for cardiac history: 31.83 (1.82, 555.81), **p=0.018.**

^3^ If adjusting for a history of falls instead of cognition, the results were as follows.

Aβ PET: 35.56 (2.08, 606.68), **p=0.014.** Tau PET: 13.09 (0.36, 466.22), p=0.158. n=195.

Aβ remained significant if also adjusting for cardiac history: 29.55 (1.71, 509.27), **p=0.020**

**STable 6B.** Multivariable logistic regression analysis with instability during 360° turning and dual-tasking as the dependent variable in cognitively healthy older adults **without a history of falls**.

Each model included both Aβ and tau, and all models were adjusted for age, sex, stroke/TIA and education.

|  | Turning 360° in standing- being unstable while dual tasking (coded 1) | | |
| --- | --- | --- | --- |
|  | Original model | Sensitivity analysis  (excl. 2 outliers tau-PET) ^1^ | Sensitivity analysis  (excl. 2 outliers tau-PET + adjusting for cognition) ^2,^ |
|  | n=213 | n=211 | n=211 |
|  | OR (95 % CI)  p-value | OR (95 % CI)  p-value | OR (95 % CI)  p-value |
| Aβ PET, SUVR | 6.93 (0.50, 95.08)  p=0.147 | 6.90 (0.50, 94.33  p=0.148 | 8.18 (0.56, 117.86)  p=0.123 |
| Tau PET, SUVR | 28.80 (1.33, 731.87) **p=0.042** | 25.85 (0.89, 749.28) p=0.058 | 29.223 (0.97, 873.35  p=0.052 |

*Note.* TIA = transient ischemic attack; OR = odds ratio; CI = confidence interval; Aβ = amyloid-β; PET = positron emission tomography;

SUVR = standardized uptake value ratio. Bolded p-values are statistically significant.

In this subsample, 103 out of 213 (48.4%) were unstable while dual-tasking, and 54 (25.8%) reported a fear of falling.

^1^ If also adjusting for cardiac history, the results were as follows. Aβ PET 6.90 (0.50, 94.33), p=0.148,

Tau PET: 25.85 (0.89, 749.28), p=0.058, n=211.

^2^ If adjusting for comfortable gait speed instead of cognition, the results were as follows. Aβ PET: 6.94 (0.50, 95.87), p=0.148,

Tau PET: 24.38 (0.80, 735.09), p=0.066. n=210. Tau remained insignificant if also adjusting for cardiac history: 23.33 (0.77, 706.85), p=0.070.

**STable 6C.** Multivariable logistic regression analysis with instability during 360° turning and dual-tasking as the dependent variable in cognitively healthy older adults **without fear of falling and without a history of falls**.

Each model included both Aβ and tau, and all models were adjusted for age, sex, stroke/TIA and education.

|  | Turning 360° in standing- being unstable while dual tasking (coded 1) | | |  |
| --- | --- | --- | --- | --- |
|  | Original model | Sensitivity analysis  (excl. 2 outliers tau-PET) ^1^ | Sensitivity analysis  (excl. 2 outliers tau-PET + adjusting for cognition) ^2,^ | |
|  | n=155 | n=153 | n=153 | |
|  | OR (95 % CI)  p-value | OR (95 % CI)  p-value | OR (95 % CI)  p-value | |
| Aβ PET, SUVR | 122.31 (3.91, 3817.77)  **p=0.006** | 120.53 (3.87, 3750.50  **p=0.006** | 139.28 (4.24, 4568.13)  **p=0.006** | |
| Tau PET, SUVR | 16.39 (0.25, 1051.03)  p=0.188 | 14.57 (0.18, 1125.80)  p=0.227 | 16.93 (0.20, 1368.80)  p=0.207 | |

*Note.* TIA = transient ischemic attack; OR = odds ratio; CI = confidence interval; Aβ = amyloid-β; PET = positron emission tomography;

SUVR = standardized uptake value ratio. Bolded p-values are statistically significant.

In this sub-group, 70 out 155 (45.2%) were unstable while dual-tasking.

^1^ If also adjusting for cardiac history, the results were as follows. Aβ PET 115.73 (3.69, 3628.46), p=0.007,

Tau PET: 12.51 (0.15, 984.78), p=0.257, n=153.

^2^ If adjusting for comfortable gait speed instead of cognition, the results were as follows. Aβ PET: 119.50 (3.86, 3699.10), p=**0.006,**

Tau PET: 13.42 (0.15, 1161.33), p=0.254. n=152. Aβ remained significant if also adjusting for cardiac history: 115.90 (3.66, 3661.29), **p=0.007.**
